# Robot-Assisted Radical Prostatectomy by the Hugo Robotic-Assisted Surgery (RAS) System and the da Vinci System: A Comparison between the Two Platforms

**DOI:** 10.3390/cancers16061207

**Published:** 2024-03-19

**Authors:** Hsien-Che Ou, Lucian Marian, Ching-Chia Li, Yung-Shun Juan, Min-Che Tung, Hung-Jen Shih, Chin-Po Chang, Jian-Ting Chen, Che-Hsueh Yang, Yen-Chuan Ou

**Affiliations:** 1 Department of Urology, Kaohsiung Medical University Chung-Ho Memorial Hospital, Kaohsiung 807, Taiwan; 1120273@mail.kmuh.org.tw (H.-C.O.); 850144@mail.kmu.org.tw (C.-C.L.); 840066@mail.kmu.org.tw (Y.-S.J.); 2 Grade Institute of Clinical Medicine, College of Medicine, Kaoshiung Medical University, Kaoshiung 807, Taiwan; 3 Department of Urology, “Pius Brînzeu” County Emergency Clinical Hospital, 300723 Timisoara, Romania; 1527952@cch.org.tw; 4 Department of Urology, School of Medicine, College of Medicine, Kaoshiung Medical University, Kaoshiung 807, Taiwan; 5 Division of Urology, Department of Surgery, Tungs’ Taichung MetroHarbor Hospital, Taichung 435, Taiwan; t1142@ms.sltung.com.tw; 6 Division of Urology, Department of Surgery, Changhua Christian Hospital, Changhua 500, Taiwan; 142893@cch.org.tw (H.-J.S.); 63569@cch.org.tw (C.-P.C.); 7 Division of Urology, Department of Surgery, Yuanlin Christian Hospital, Changhua 510, Taiwan; 1520014@cch.org.tw

**Keywords:** male, prostatic neoplasms/surgery, prostatectomy/methods, robotic surgical procedures/methods, robotics/methods

## Abstract

**Simple Summary:**

This study reports on a comparison between the Hugo RAS system and the da Vinci (DV) system in robot-assisted radical prostatectomy. The parameters consisted of intraoperative anastomosis and overall console time, functional outcomes, and oncological outcomes. We found that, although the anastomosis time of the urinary bladder and urethra was lengthened, the overall console time was not affected. In short-term functional and oncological outcomes, these two parameters were not compromised in the Hugo RAS group. Based on our results, the DV system could facilitate swift adaptation to the Hugo RAS system. Importantly, the functional and oncological outcomes would not be compromised in the process of adapting to the Hugo RAS system.

**Abstract:**

Objective: In a previous study, we proved that an experienced urologist is more likely to adapt to the Hugo RAS system. Based on this, we further examine various parameters in this study. Parameters included in this study consisted of console time, functional outcomes, and oncological outcomes. Materials and Methods: A total of 60 patients who underwent robot-assisted radical prostatectomy (RARP) performed by a single surgeon using the da Vinci (DV) system (n = 30) or the Hugo RAS system (n = 30) between March 2023 and August 2023 were included in the analysis. The intraoperative operative time was categorized into vesicourethral anastomosis time and overall console time. Functional and oncological outcomes were documented at the 1st and 3rd postoperative months. Parametric and non-parametric methods were adopted after checking skewness and kurtosis, and an α value of 5% was used to determine the significance. Results: The vesicourethral anastomosis time was significantly lengthened (Hedge’s g: 0.87; 95% confidence interval (CI): 0.34–1.39; J factor = 0.987). However, the overall console time was not affected. The functional (postoperative 3rd month: *p* = 0.130) and oncological outcomes (postoperative 3rd month: *p* = 0.103) were not significantly different. We also found that the adverse effect on surgical specimens and positive surgical margins was not affected (*p* = 0.552). Conclusion: During the process of adaptation, although intricate motions (such as the vesicourethral anastomosis time) would be lengthened, the overall console time would not change remarkably. In this process, the functional and oncological outcomes would not be compromised. This encourages urologists to adopt the Hugo RAS system in RARP if they have previous experiences of using the DV system, since their trifecta advantage would not be compromised.

## 1. Introduction

Since 1985, when the first robotic surgery was conducted, robotic technology has blossomed rapidly. In the past twenty years, surgery has evolved from being an open operation to being minimally invasive. Since then, almost all technologies related to surgery have been developed based on the latter procedure, including the robotic system. In the beginning, robotic platforms were created to allow surgeons to perform operations remotely in battlefields. At first, patients were hesitant to receive robot-assisted surgeries due to the expensive charges. As advertisement increased and related insurance policies became favorable, patients started to take robotic surgeries into consideration [1]. In the future, developments in artificial intelligence and 5G technology are expected to further facilitate the innovation of robotic surgeries and speed up the learning curve for surgeons [2].

In the age of open surgery, the surgical fields available to urologists were limited and blood loss during surgery was massive. Hence, the preservation of functional ability, including continence and potency, was considered a low priority. With the inception of minimally invasive surgical procedures, many remarkable advancements have been achieved in the realm of radical prostatectomy, including laparoscopic radical prostatectomy and robot-assisted radical prostatectomy (RARP). RARP, compared with open radical surgery, could greatly decrease the postoperative complications and estimated blood loss [3]. Applying robotic systems in radical prostatectomy could benefit patients by decreasing operative time, blood loss, transfusion rates, hospital stays, and complication rates [3]. In the abovementioned parameters, the advantage of decreasing the transfusion rate has been particularly consistent among the published studies (I^2^ = 0). Regarding oncological outcomes, RARP decreased the biochemical recurrence of prostate-specific antigens (PSAs) ≥ 0.2 ng/mL. Regarding functional outcomes, only erectile function after RARP was recovered; continence was not recovered. This could be attributed to the higher rate of neurovascular bundle preservation in RARP. However, regarding continence recovery, erectile recovery, or neurovascular bundle preservation rates, there is much heterogeneity among the published literature [3].

While the da Vinci (DV) system (Intuitive Surgical Inc., Sunnyvale, CA, USA) has monopolized robot-assisted surgeries for nearly two decades [4,5], the release of its key patents has led to the development of other robotic surgery platforms, including the Hugo RAS system (Medtronic, Minneapolis, MN, USA) [6]. Previous robotic technologies improved the approach to prostate cancer surgery, with enhanced precision and reduced invasiveness. Most importantly, it maximally optimizes the functional outcomes for patients [7,8,9]. However, as with many emerging medical technologies, its trustworthiness and comparative efficacy always need to be examined. This is particularly important in Asia because around 60% of the global population lives there, and there is much variation in economic and healthcare environments as compared to other continents. In addition to basic healthcare services, economic issues needed to be considered when obtaining informed consent from patients, especially in developing countries [10].

This research paper aims to compare the surgical and functional outcomes of the Hugo RAS system with that of the DV system in RARP, evaluating the trustworthiness and clinical utility of this new platform. By examining the key parameters and analyzing the available evidence, we hope that this study could inform both urologists and patients about the potential benefits and limitations of performing RARP using the Hugo RAS system. Until the present, only one study has compared the Hugo RAS system with the DV platforms in RARP, and it is a European-population-based study [11]. Thus, this study represents the first Asian study comparing the surgical and functional outcomes of these two platforms, offering valuable insights into the efficacy and safety of this new robotic surgery platform among the Asian population.

## 2. Materials and Methods

This retrospective study recruited patients diagnosed with adenocarcinoma of PCa between March 2023 and August 2023. Participants were informed of RARP and discussions were undertaken in accordance with the PCa guidelines published by the National Comprehensive Cancer Network [12]. Diagnosis of PCa was carried out either by trans-rectal ultrasound (TRUS)-guided biopsy or magnetic resonance image (MRI)/TRUS fusion biopsy. All malignant results would be confirmed by peer review by pathologists. Men with localized PCa more than intermediate risk would regularly have their MRI and whole-body bone scan assessed for the preoperative stage and to rule out distant metastasis. Participants mainly consisted of men with localized PCa and metastatic PCa; patients receiving any neoadjuvant therapies before RARP were excluded from the analysis. Surgical platforms, either the DV system or the Hugo RAS system, were freely selected by the patients after fully explanation. RARP procedures, using either the Hugo RAS system or the DV system, were all carried out by the same expert, Yen-Chuan Ou (>3000 cases). In our practice, RARP was arranged to take place four–six weeks after TRUS-guided biopsy or MRI/TRUS fusion biopsy; the lymphadenectomy was conducted along with RARP.

Assessments included age, body mass index (BMI), PSA level, percentage of positive cores, clinical staging, pathology Gleason score, and pathological T stage. Specimen weight and tumor percentage were also analyzed [13,14,15]. The PI-RADS score was interpreted according to the protocol issued by the American College of Radiology (version 2.1; available at: https://www.acr.org/-/media/ACR/Files/RADS/PI-RADS/PIRADS-V2-1.pdf (accessed on 1 March 2023)).

Intraoperative assessments included the total console time and vesicouethral anastomosis time. In surgical specimen, positive surgical margins (PSM) were deemed to be an adverse effect [12]; this was confirmed through peer review by pathologists. Other recordings included bilateral neurovascular bundle (NVB) preservation, blood loss amount, blood transfusion, and surgical conversion rate [15,16,17]. Complications were documented and stratified according to the Clavien–Dindo classification [18]. In oncological assessment, the first serum PSA would be investigated at the third month after RARP. Nadir at our facility was determined to be 0.008 ng/mL. Functional outcomes were measured by continence and potency recovery rate at 1 and 3 months after RARP. For potency, we used the International Index of Erectile Function (IIEF)-5 to compare the preoperative and postoperative differences. Preoperative dysfunction would be excluded from analysis. We assessed continence by recording the pads they used after RARP; 0 pads–1 pad used per day was taken to indicate continence recovery.

The statistical analysis was conducted by employing the parametric or non-parametric methods according to their skewness and kurtosis. The α value of 5% was used to judge the significance, and significant parameters would be further analyzed with their effective size. These recorded data were maintained in Microsoft Excel (Version 2016, Microsoft Corp., Redmond, WA, USA) and analysis was performed by using R (R Core Team (2021). R is a language and environment for statistical computing (R Foundation for Statistical Computing, Vienna, Austria; URL: https://www.R-project.org/ (accessed on 3 December 2023)).

## 3. Results

Among the 60 men recruited, 45 (75%) were diagnosed with TRUS-guided biopsy and the other 15 (25%) men were diagnosed with MRI/TRUS fusion biopsy. They were evenly divided into Group 1 (DV group; n = 30) and Group 2 (Hugo RAS group, n = 30). The preoperative parameters were documented in Table 1, and there were no remarkable differences among them. In intraoperative assessments (Table 2), no intraoperative conversions were encountered. Neither intraoperative blood transfusion nor complications occurred. We noticed that the time spent on vesicourethral anastomosis using the Hugo RAS platform would be significantly longer than that using the DV system; meanwhile, the overall console time remained the same. The Hedge’s g of anastomosis was 0.87 (95% confidence interval (CI): 0.34–1.39; J factor = 0.987), and the odds ratio from the univariable logit regression was 0.79 (95% CI: 0.68–0.93 (reference group: Hugo RAS group); *p* = 0.003). Multivariable logit regression was not possible to establish due to the absence of the required dependent variables.

Similar conditions were observed for the total console time, blood loss, NVB preservation, pathological stage, and resected prostate weights. Neither intraoperative conversion nor blood transfusion occurred during the surgery. The postoperative parameters are recorded in Table 3. Regarding the surgical adverse effects, the PSM rate was also similar between the two groups. There were no differences in the total dissected lymph nodes (median = 7 in Group 1; median = 8 in Group 2) nor positive lymph nodes (median = 3 in Group 1; median = 3 in Group 2). For the functional outcomes, no one was excluded due to preoperative erectile dysfunction. Postoperative 1- and 3-month potency recovery rates and continence recovery rates remained the same between the two groups. Aside from that, the PSA nadir rate investigated in the third month after RARP was alike between the two groups. The 60 patients were basically all on a 7-day admission protocol, and urethral catheters were removed on the 7th day before their discharge. Discharge was arranged once micturition was observed after removing their urethral catheters. Since slight leakage was noticed after vesciourethral anastomosis, there were two men who had their urethral catheters indwelled for the 8th and 9th days, respectively. Their urethral catheters were all removed with successful self-micturition, and neither urinoma nor urine accumulation outside the urinary bladder was noted in the sonography at the clinics. There were only three patients noted to have ileus after RARP, but they were successfully managed with conservative medical treatments and their admissions were not prolonged as a result. Otherwise, no complications were observed.

## 4. Discussion

Until the present, over 3500 articles discussing robot-assisted surgeries in the urological field have been published [19]. Among all commercially available robotic systems, the Revo-i Surgical Robot (Meerecompany, Seoul, Republic of Korea) system achieved 12 degrees of freedom in its robotic arms, surpassing all other robotic platforms [19]. The only human trial [20] in the literature reported that, out of 17 men, only 1 (6%) had a PSA that did not drop to the nadir at the third month after RARP; however, the trial was small in size and did not provide a comparison to the DV system. In Taiwan, before the introduction of the Hugo RAS system, there were only two platforms available, which were Senhance surgical system (Asensus Surgical, Inc., Durham, NC, USA) and the DV system. In their designs, the maximal numbers of robotic arms were the same, but there were fewer degrees of freedom in the robotic arms in the Senhance surgical system [19]. In one article comparing the perioperative outcomes of these two platforms [21], while the functional and oncological outcomes were similar in these two platforms, the Senhance surgical system could provide advantages in the medical costs for patients. Recently, the Hugo RAS system was introduced into Taiwan for less than a year. In experiences from Europe [11], the total console time, the PSM, and continence recovery seemed to be similar between the DV system and the Hugo RAS system according to multivariable models.

In this study, we found that the vesicourethral anastomosis time was longer in RARP using the Hugo RAS system than it was using the DV system. In comparison, the arms’ degree of freedom of the Hugo RAS system was the same with the DV system, and they both could provide 7 degrees of freedom in their robotic arms [19]. The Hugo RAS system adopted independent arm carts and the range of motion, to some degree, was affected by the positioning of the arm carts [19,22]. In this way, the ports’ malposition in our initial 30 cases [23] with the Hugo RAS system would prolong the manipulation, especially the delicate suturing of the vesicourethral anastomosis. Another source of difference might be attributed to their console. The DV system adopts a closed console headset, while the Hugo RAS system has a console headset that is open to the environment. In the published literature, there is an opinion that such a design could increase intraoperative errors and decrease operative efficiency [19]. However, in our analysis, the overall operative time was not affected. This implied that, although some certain surgical steps (especially steps needing sophisticated manipulation) might be prolonged due to the use of unfamiliar platforms, little change occurs in the overall surgical console time. Aside from this, in our prior study, a quick adaptation to the Hugo RAS system could be expected among people with previous experiences of using the DV system [23]. This also contributed to the minimal change in the overall console time, since great improvement in various intraoperative parameters was observed in our first 12 cases with the Hugo RAS system. In the future, more and more technologies will be incorporated into robotic surgical platforms, such as haptic feedback [19], and this will make the adaption quicker.

Although our study consisted of fewer patients than [11], in addition to having inferior statistical power, the overall console time of our data was a lot lower than the that in [11]. According to their data [11], a new Hugo RAS system user could achieve RARP within approximately 30 min after their first 80 cases. In our data, the overall console time seemed to less than theirs, by sixty minutes, and only 30 cases were performed by our operator. This difference implies that a multivariable model could be further established to control how many DV system RARP cases were performed before their initial cases with the Hugo RAS system.

In other intraoperative parameters, we observed that our data show blood loss to be lower than that reported in [11]. However, higher prevalence of performing neurovascular bundles was observed in their study, and this might contribute to the different functional outcomes in the first 3 months after RARP. The preservation of neurovascular bundles and adequate urethral stumps was essential for functional recovery. This depended not only on the anatomical features but also surgical techniques. This was also one of the reasons why robotic platforms have outpaced the open method in radical prostatectomy, since it allows more delicate preservation of anatomy. In the previously published study [11], functional outcome recovery seemed to be better than that in our study. Otherwise, this might be another type-2 error, resulting from the small sample size in our study. Thus, we will precisely analyze the results of performing neurovascular bundles preservation and functional outcome recovery in the future, with an expanded sample size.

Regarding the postoperative continence and potency rate in 3 months, it seemed that patients after RARP with the Hugo RAS system had a better recovery rate, increased by around 20% in comparison with that of patients after RARP with the DV system. However, this observation did not reach statistical significance. In our previous report, RARP in localized PCa featured a high postoperative trifecta rate [24]. After 12-month follow-ups, the continence recovery rate was 97%. The potency recovery and biochemical-free rates were 87% and 94.6%, respectively. Overall, the trifecta rate reached 82% [24]. In this study, we compared the functional outcomes (potency and continence) of the Hugo RAS system with those of the DV system in the short term after RARP. PSA nadir was included in oncological parameters.

Regarding the PSM, we observed that there was tendency for the PSM to be lower after RARP with the Hugo RAS system. In our first 300 cases of RARP with the DV system, the PSM rate ranged from 15% to 30% [24], and this was comparable to our data in this study. Since the PSM rates in both groups were in the range of 15% to 30%, this tendency might be explainable as an insignificant between-group difference. Putting functional outcomes and oncological outcomes together, we found that, even though the Hugo RAS system was new to a DV system user, these two outcomes would not be compromised in their early experiences with the Hugo RAS system. Meanwhile, the rate of PSM would not increase. These results were in line with our observation that quick adaptation to the Hugo RAS system could be expected in a DV system user [22] and could give confidence to a beginner in using the Hugo RAS system. However, the actual trifecta rate of RARP with the Hugo RAS system in the long run needs to be discussed, with an expanded study and longer surveillance in the future.

Although the experiences from the DV system could help early adaptation to the Hugo RAS system [22], surgeons still needed time to become accustomed to some different devices on the Hugo RAS system, such as hand controls and arm carts’ degrees of freedom [25]. We mentioned that the vesicourethral anastomosis time was longer in the Hugo RAS group in this study. In our experience, the arms’ range of motion of the Hugo RAS system was somewhat different from the DV system, and the motion of the wrist would determine the fluency. In this way, the different hand controls, being unfamiliar to new operators, would naturally lengthen this step. However, the overall operative time was not affected. That implied that, although the process of adapting to the different hand controls would lengthen certain surgical steps, little change in the overall operative console time would occur. Regarding this observation, we will expand the sample size so as to have enough statistical power to draw this conclusion in the future.

It is important to introduce the nuanced aspects of the Hugo RAS system, since it comes with several merits, including the console-interface-improving ergonomics and different arm carts, which allow port placements according to the surgeons’ preference [23]. Several previously published studies have highlighted these ergonomic benefits, especially in mitigating surgeon discomfort, compared to the bury-in position of the DV platform [23,26,27]. The discomforts immediately after using the DV platform are more significant for surgeons with a high BMI (26–30 kg/m^2^) [27]. Thus, such surgeons would benefit more if they successfully adapted to the Hugo RAS system. Currently, the transferability between platforms and the effectiveness of simulator training could significantly speed up the learning curve for surgeons [23,28]. There were still some disadvantages in the Hugo RAS system. The separated arm carts system of the Hugo RAS may cause more spatial constraints in operating rooms than the DV system. Another disadvantage is the limited available assists from the robotic platform. The current selection of instruments that are compatible with the Hugo RAS are fewer than those for the DV system, and some auxiliary tools such as Tile-Pro sonography and the indocyanine green enhancement techniques are not yet available in the Hugo RAS system [29,30].

The major limitation of this study is statistical power. Since this study was just a preliminary study to detect the possible differences between the Hugo RAS system and the DV system, an expanded study, including more critical conditions in RARP, needs to be designed in the future [31,32,33,34,35]. Another deficit of this article is that, since no sufficient meaningful variables were identified, we were unable to establish a multivariable model with a maximum likelihood estimation to discuss each parameter independently. Thus, more meaningful variables need to be explored to allow a sophisticated analysis to be performed. Another limitation is that our participants mainly consisted of low- and intermediate-risk groups. Thus, the PSM rate and oncological outcomes might be elevated after including high-risk patients. Another limitation is that this was a single-center, single-surgeon, retrospective study. Instead of trying to apply our results universally, this initial analysis was more of an internal validation study. In this way, we need to expand our study by including more surgeons with different practices and from different hospitals.

Another issue that has not been addressed in this study is the economic aspect. In the early development of robot-assisted surgeries, high medical expenses are always important issues for discussion, especially in Asia. In Taiwan, due to the special public healthcare insurance system, patients are more aware of their medical expenses. In Taiwan, the purchase price of the Hugo RAS system was much lower than that of the DV system. In this way, the economic costs might be another advantage of the Hugo RAS system over the DV system. However, in this study, most of the participating patients were not willing to provide their actual expenses upon discharge for privacy reasons; therefore, the economic aspects could not be compared in this analysis. The coming study may be able to address this viewpoint.

## 5. Conclusions

Our previous study and the present one have validated RARP with the Hugo RAS system as a safe and feasible alternative to the DV system. Despite longer operation times in vesicourethral anastomosis, RARP with the Hugo RAS system has been demonstrated to have comparable outcomes, such as functional outcomes, oncological outcomes, and surgical adverse effects. These findings were encouraging, a future discussion with an expanded sample size would be worthwhile to achieve sufficient statistical power. More meaningful variables could be detected by including more critical parameters. In this way, the differences between the Hugo RAS system and the DV system could be discussed meticulously.

## Figures and Tables

**Table 1:** Preoperative parameters.

	Group 1 (n = 30)	Group 2 (n = 30)	*p*-Value
Age (years old; median (IQR ^a^))	67.50 (11.50)	66.50 (10.00)	0.473 §
BMI ^b^ (kg/m^2^; median (IQR ^a^))	26.00 (4.34)	25.79 (4.26)	0.584 §
PSA (ng/mL; median (IQR ^a^))	9.46 (9.53)	8.81 (7.66)	0.496 §
ASA ^c^ grade (n; %)			1.000 †
II	14 (46.7%)	14 (46.7%)	
III	16 (53.3%)	16 (53.3%)	
DRE ^d^ (n; %)			1.000 †
Positive	8 (26.7%)	9 (30%)	
Negative	22 (73.3%)	21 (70%)	
PI-RADS score (n; %)			0.458 †
Grade 3	19 (63.3%)	18 (60%)	
Grade 4	9 (30%)	7 (23.3%)	
Grade 5	2 (6.7%)	5 (16.7%)	
Biopsy Gleason score (n; %)			0.800 †
≤3 + 3	16 (53.3%)	17 (56.7%)	
3 + 4	8 (26.7%)	6 (20%)	
4 + 3	3 (10%)	3 (10%)	
4 + 4; 3 + 5; 5 + 3	1 (3.3%)	3 (10%)	
4 + 5; 5 + 4; 5 + 5	2 (6.7%)	1 (3.3%)	
Maximal percentage of malignancy on positive cores (%; median (IQR ^a^))	0.13 (0.16)	0.10 (0.13)	0.151 §
Clinical T stage (n; %)			0.206 †
2a	13 (43.3%)	16 (53.3%)	
2b	11 (36.7%)	5 (16.7%)	
2c	6 (20%)	9 (30%)	

^a^ Interquartile range; ^b^ body mass index; ^c^ American Society of Anesthesiologists; ^d^ digital rectal exam; § Mann–Whitney U test; † Fisher’s exact test;

**Table 2:** Intraoperative parameters.

	Group 1 (n = 30)	Group 2 (n = 30)	*p*-Value
Vesicourethral anastomosis (min; median (IQR ^a^))	16.00 (6.00)	19.50 (6.25)	0.003 § **
Console time (min; median (IQR ^a^))	133.00 (36.00)	146.50 (36.50)	0.130 §
Neurovascular bundles preservation (n; %)			0.789 †
Not performed	12 (40.0%)	10 (33.3%)	
Performed	18 (60%)	20 (66.7%)	
Blood loss (mL; median (IQR ^a^))	200.00 (177.50)	187.50 (242.50)	0.812 §

^a^ Interquartile range; § Mann–Whitney U test; † Fisher’s exact test; ** *p* < 0.005

**Table 3:** Postoperative parameters and postoperative complications according to the Clavien–Dindo classification.

	Group 1 (n = 30)	Group 2 (n = 30)	*p*-Value
Pathological stage (n; %)			0.759 †
2a	17 (56.7%)	16 (53.3%)	
2b	8 (26.7%)	8 (26.7%)	
2c	1 (3.3%)	3 (10%)	
3a	4 (13.3%)	3 (10%)	
Resected prostate weight (gram; median (IQR ^a^))	41.00 (19.00)	40.00 (16.50)	0.953 §
Tumor percentage in pathology (%; median (IQR ^a^))	8.59 (8.28)	6.45 (10.63)	0.355 §
Surgical margins (n; %)			0.552 †
Positive	9 (30%)	6 (20%)	
Negative	21 (70%)	24 (80%)	
Angiolymphatic invasion (n; %)			0.748 †
Positive	5 (16.7%)	7 (76.7%)	
Negative	25 (83.3%)	23 (23.3%)	
Perineural invasion (n; %)			0.567 †
Positive	7 (76.7%)	10 (33.3%)	
Negative	23 (23.3%)	20 (66.7%)	
Potency recovery after 1 month (n; %)			1.000 †
Positive	6 (20.0%)	7 (23.3%)	
Negative	24 (80.0%)	23 (76.7%)	
Potency recovery after 3 months (n; %)			0.180 †
Positive	16 (53.3%)	22 (73.3%)	
Negative	14 (46.7%)	8 (26.7%)	
Continence recovery after 1 month (n; %)			0.761 †
Positive	6 (20%)	8 (26.7%)	
Negative	24 (80%)	22 (73.3%)	
Continence recovery after 3 months (n; %)			0.103 †
Positive	16 (53.3%)	23 (76.7%)	
Negative	14 (46.7%)	7 (23.3%)	
PSA nadir after 3 months (n; %)			1.000 †
Yes	19 (63.3%)	18 (60%)	
No	11 (36.7%)	12 (40%)	
Postoperative e complications (n, %)			
Leakage at vesciourethral anastomosis			
Clavien–Dindo grade 1	2 (6.7%)	0 (0%)	N/A ^b^
Postoperative ileus			N/A ^b^
Clavien–Dindo grade 1	1 (3.3%)	1(3.3%)	
Clavien–Dindo grade 2	1(3.3%)	0 (0%)	

^a^ Interquartile range; ^b^ not applicable; § Mann–Whitney U test; † Fisher’s exact test

## Data Availability

The data can be shared upon request.
